# Midwives’ and Other Perinatal Health Workers’ Perceptions of the Black Maternal Mortality Crisis in the United States

**DOI:** 10.1111/jmwh.13433

**Published:** 2022-11-22

**Authors:** Melva Craft‐Blacksheare, Peggy Kahn

**Affiliations:** ^1^ School of Nursing University of Michigan‐Flint Flint Michigan; ^2^ Department of Political Science University of Michigan‐Flint Flint Michigan

**Keywords:** Black maternal mortality, midwives, race, social determinants of health

## Abstract

**Introduction:**

This study aimed to identify how perinatal health workers, especially midwives, explained US Black maternal mortality and morbidity and what ameliorative measures they suggested across categories of primary social determinants, health care access, and provider practices.

**Methods:**

Using a mixed closed‐ended and open‐ended researcher‐designed exploratory survey, 227 perinatal health workers responded to a series of questions probing views of causation and strategies for improvement. The closed‐ended responses were summarized. Open‐ended responses were analyzed using basic categorical and thematic coding.

**Results:**

Perinatal health workers’ responses prominently identified racism as a cause of Black maternal morbidity and mortality, and their recommendations ranged across levels of social determination of health.

**Discussion:**

Results suggest that the views of perinatal health workers, the majority of whom were midwives, are complex and correspond to the problems and solutions identified in the research literature. Midwives and other perinatal health workers are well positioned to help center health equity in perinatal care, through both clinical practice and policy advocacy.

## INTRODUCTION

An extensive literature documents and analyzes excessive Black maternal mortality in the United States. In the United States, the 2020 maternal mortality rate, the number of deaths per 100,000 live births including maternal death during pregnancy or within 42 days of termination of pregnancy from a cause related to or aggravated by pregnancy, was 23.8 per 100,000 live births. The maternal mortality rate for non‐Hispanic Black women was 55.3 deaths per 100,000 live births, 2.9 times the rate for non‐Hispanic white women (19.1), and higher than the rate for Hispanic women (18.2).^1^ Racial and ethnic disparities with particularly alarming outcomes for Black women have persisted over time.^2^ Measures of pregnancy‐related deaths (pregnancy‐related death within one year of the end of the pregnancy) and severe maternal morbidity also reveal stark racial and ethnic disparities.^3^, ^4^

**QUICK POINTS**

✦ This analysis is one of the first to describe the views of midwives and other perinatal health workers regarding Black maternal mortality, surveying a convenience sample (N = 227).
✦ Midwives and other perinatal health workers attribute excessive Black maternal mortality to negative primary social determinants of health, compromised access to health care, and problematic provider practices. They correspondingly recommend solutions across these 3 levels of determinants.
✦ At the level of provider practices, midwives and other perinatal health workers recommend attention to the workforce: more racial and cultural sensitivity training, more recruitment of providers of color, and more midwives in the birth workforce.
✦ Midwives and other perinatal health workers also suggest increased modes of service delivery (eg, multispecialty provider groups, birth centers), more evidence‐based practice, and more careful listening to clients.
✦ Responses to this survey suggest midwives and others are positioned to become institutional leaders in centering health equity.

Research on Black maternal morbidity and mortality reflects a shift in health research away from race as a biological determinant toward race as a social construct and away from individual behavior independent of social context toward structural determinants.^5^, ^6^ Researchers have attributed high Black maternal death and morbidity rates to the association of race with negative social determinants of health, the environment in which people undertake everyday life activities, and the effects on health and quality of life. The social and economic consequences of racism in everyday life experiences, social structures, and health care institutions have been linked to poor maternal and infant outcomes.^7^, ^8^ Researchers have also identified biological pathways through which racism and other forms of toxic stress directly impact maternal and infant health.^9^

No previous studies have broadly explored midwives’ and other perinatal health workers’ perspectives on Black maternal mortality. Previous studies of US midwives’ attitudes, perceptions, and beliefs have focused elsewhere, for example, on newborn screenings,^10^ men in midwifery,^11^ infant safe sleep,^12^ and planned home birth.^13^ Almanza et al identified the motivations of 7 midwives of color—their strong commitments to racially concordant care, racial justice, and physically and emotionally safe care—associated with an African American‐owned community birth center.^14^ Midwives, emphasizing person‐centered care to promote healthy pregnancy and reduce medical interventions, are well positioned to understand not only individual clients’ health histories and the physiology and management of pregnancy and childbirth but also clients’ environmental conditions including racist structures and practices. Although attending only 9.9% of births in the United States,^15^ midwives can offer insights into causal factors, attest to current practices, and offer suggestions to improve outcomes. This study, therefore, aimed to identify how midwives and other perinatal health care workers of various ethnicities across a variety of settings explain causes of and remedies for excessive US Black maternal mortality and morbidity.

## METHODS

This exploratory descriptive research, approved by the University of Michigan‐Flint Institutional Review Board, posed closed‐ended and open‐ended questions to perinatal health workers in 2019 to ascertain their assessment of factors contributing to excessive Black maternal mortality and recommendations for reducing poor perinatal outcomes. The survey elicited descriptive and qualitative information.

Two Black perinatal health nurses, a certified nurse‐midwife (CNM) and a certified women's health nurse practitioner, each with a doctoral degree, developed the survey. The survey questions were informed by their own urban maternal nursing experiences and conversations in professional networks and by their reading of the nursing and biomedical literature. The survey was not piloted or reviewed by additional parties. The questionnaire did not force responses and was structured to discover midwives’ and other perinatal health workers’ awareness of excessive Black maternal mortality, their general views of factors contributing to high Black maternal mortality, their direct professional observations regarding perinatal care, and their suggestions for improving perinatal outcomes, especially those of Black women.

The survey included 7 initial questions eliciting respondents’ characteristics: race and ethnicity, gender, age, professional position and years of experience, ethnicity of clients, and type of practice community (urban, rural, suburban) (see Table 1); 1 closed‐ended question about awareness of maternal mortality rates (see Table 2); 3 closed‐ended questions about Black maternal mortality (Table 3); 7 closed‐ended questions about clinical practices with a 7‐point Likert scale ranging from 1 (strongly disagree) to 7 (strongly agree) (Table 4); and 7 open‐ended questions probing participants’ general perceptions about Black maternal mortality (Table 5).

**Table 1:** Demographic and Other Characteristics of Participants

Characteristic	n^a^ (%)
**Race and ethnicity**	194
Asian American	4 (2.06)
Black or African American	29 (14.95)
Hispanic or Latinx American	2 (1.03)
Middle Eastern	1 (0.52)
American Indian	1 (0.52)
Pacific Islander	0
White or European American	150 (77.32)
Other	5 (2.58)
Prefer not to answer	2 (1.03)
**Gender**	191
Female	189 (98.95)
Other	2 (1.05)
**Profession**	193
Certified nurse‐midwife	129 (66.84)
Nurse practitioner	12 (6.22)
Certified professional midwife	9 (4.66)
Student midwife	9 (4.66)
Doula	8 (4.15)
Direct entry midwife	3 (1.55)
Certified midwife	1 (.52)
Obstetrician‐gynecologist	1 (.52)
Other	21 (10.88)
**Years of practice**	193
0‐4	66 (34.19)
5‐10	38 (19.69)
11‐15	17 (8.81)
>15	72 (37.31)
**Age**	192
20‐26	3 (1.56)
27‐39	63 (32.81)
40‐49	55 (28.65)
50‐59	29 (15.10)
60+	42 (21.86)
**Area of practice**	188
Urban	69 (36.70)
Suburban	45 (23.94)
Equal mixture (urban and suburban)	40 (21.28)
Rural >50%	34 (18.09)

^a^ The variable number of responses reflects uneven responses to survey questions.

**Table 2:** Awareness of Increasing Maternal Mortality Rates in the United States

**Total (N = 195)**	**n (%)**
Very aware	175 (90)
Somewhat aware	20 (10)
Not at all aware	0

**Table 3:** Awareness of Black Maternal Mortality (Yes, No Questions)

**Question** **^a^**	**Yes n (%)**	**No n (%)**
Do you think the maternal mortality/morbidity rate is relatively the same between white/European American women and women of color? (n = 195)	2 (1.03)	193 (98.97)
Should Black maternal mortality/morbidity be addressed on a practitioner level? (n = 174)	160 (91.95)	14 (8.05)
Senate Bill 3363 supports states using evidence‐based practices and quality improvement practices. Do you think this is a good action to address this problem? (n = 166)	152 (91.57)	14 (8.43)

^a^ The variable number of responses reflects uneven responses to survey questions.

**Table 4:** Clinical Practices (Likert Scale Questions)

**Question** **^a^**	**Strongly Disagree n (%)**	**Disagree n (%)**	**Somewhat disagree n (%)**	**Neither agree or disagree n (%)**	**Somewhat agree n (%)**	**Strongly agree n (%)**	**Agree n (%)**
As a health practitioner, I have never witnessed implicit bias during maternity care toward a woman of color (n = 172)	77 (44.77)	43 (25.00)	16 (9.30)	8 (4.65)	4 (2.33)	10 (5.81)	14 (8.14)
As a health practitioner, I sometimes notice women with Medicaid receiving a different level of care in the office and/or hospital (n = 172)	12 (6.98)	20 (11.63)	12 (6.98)	12 (6.98)	30 (17.44)	33 (19.19)	53 (30.81)
As a health care provider, I believe all women in my practice receive the same level of care from all the maternity team members (n = 172)	20 (11.63)	22 (12.79)	26 (15.11)	10 (5.81)	19 (11.05)	29 (16.86)	46 (26.74)
It is important to respect and appreciate people's diverse social identities (n = 173)	7 (4.05)	0	0	0	0	143 (82.66)	23 (13.29)
It is important that maternity care promote physical and emotional health (n = 173)	3 (1.73)	0	0	0	0	156 (90.17)	14 (8.09)
Women should have meaningful input into their decisions surrounding childbirth (n = 173)	2 (1.16)	0	0	0	0	157 (90.7)	14 (8.09)

^a^ The variable number of responses reflects uneven responses to survey questions.

**Table 5:** Responses to Open‐Ended Questions

**Question**	**Number of Responses**
What are examples of how practitioners can address the Black maternal mortality rate?	144
Why can this rate not be addressed at the personal practitioner level?	14
What would you say to your client about the Black mortality/morbidity rate?	136
Why do you think the Black mortality/morbidity rate is high?	145
What methods would you suggest/explore to decrease the Black mortality/morbidity rate?	141
Why do you think Senate Bill 3363 is a good measure?	117
Why do you think Senate Bill 3363 is not a good measure?	13

The Qualtrics online survey was placed on social media group sites of perinatal health field workers, including midwives, doulas, and lactation consultants. The group sites were not specifically oriented toward Black women or perinatal health care workers of color. Additionally, an internet link was sent to deans of 50 schools of nursing with nurse practitioner programs and clinical faculty in maternal health, encouraging further distribution to maternal health colleagues and students. Researchers cast a wide net, aware that Black people giving birth were served not only by midwives but also by doulas, nurse practitioners, and other perinatal professionals. Sample recruitment and survey completion occurred from May 2019 until December 2019.

Prior to beginning the survey, respondents were notified of the purposes of the study, institutional review board approval, and likely time for survey completion. The survey provided the contact information of the principal investigator. Informed consent was implied by survey commencement. Survey results included responses of 227 perinatal health workers. Survey responses were automatically stored on a secure password‐protected Qualtrics account. Participants were not reimbursed.

### Data Analysis

For closed‐ended and questions employing 7‐point Likert scales, number and percentage of response values for each item were calculated.

Open‐ended responses were initially coded using preexisting categories (primary social determinants, access to health care, provider practices) employing Miller and Crabtree's template qualitative analysis procedure.^16^ Primary social determinants of health, also referred to as upstream factors, were understood to refer to broad structural and community characteristics.^17^ Health care access is an intermediate determinant of health outcomes because it can mediate the impact of social determinants (and together with health care quality is critical to health outcomes). Provider practices are social constructions downstream from policy and community determinants and access to health care. Additional emerging subthemes within those preexisting categories were identified during the analysis. The 2 authors independently reviewed and analyzed the data, compared codes, and discussed the patterns and themes that emerged. The 2 authors corroborated each other's coding, the frequency with which phrases and ideas appeared, and the interpretation of phrases and sentences in the context of health care practice, to increase dependability.^18^, ^19^

## RESULTS

A total of 229 participants opened the survey online, and 227 participated. One hundred sixty‐eight respondents progressed through the entire survey. Reported results include not only responses of the 168 completers but usable data from all 227 participants. Not every respondent counted as completing the survey answered every question, as the survey instrument did not force responses. CNMs comprised 66.8% of the 193 respondents identifying their profession. Other perinatal health workers comprised smaller percentages of respondents. Of 194 participants identifying their race and ethnicity, 15% were Black, and a majority identified as white. Table 1 details demographic and other characteristics of participants.

Of 195 responses to each of the 2 questions about levels of maternal mortality, 195 (100%) indicated awareness of the increasing maternal mortality rate in the United States (Table 2), and 193 indicated knowledge that Black maternal mortality exceeded that of white women (Table 3). Most participants had witnessed implicit bias toward women of color and women insured through Medicaid within their professional experiences, with respondents evenly divided about whether all women in their practice received the same level of care from all maternity team members. Ninety five percent or more of respondents indicated that it was important for practitioners to respect people's diverse social identities, attend to clients’ physical and emotional health, and allow women to have meaningful input into decisions about their care and childbirth. A majority (91.95%) of respondents indicated that the Black maternal mortality rate should be addressed with their clients, and 14 (8.05%) indicated it should not (Table 3).

Each open‐ended question yielded a range of 14 to 145 responses (Table 3), allowing for robust coding according to the template categories and emerging themes. Template categories included primary social determinants, health care access, and provider practices. Several subthemes related to provider practices emerged in responses to these questions: the perinatal workforce, modes of perinatal service delivery, interpersonal listening and attention to individual clients, evidence‐based perinatal practices, and midwives’ and other perinatal practitioners’ roles in the clinical setting.

### Primary Social Determinants

Respondents named structural racism, white supremacy, overall socioeconomic oppression, and systemic racism as causes of excessive Black maternal mortality. While noting that socioeconomic factors were often related to race, they also identified racism as operating across the class structure to the detriment of Black women. They suggested that childbearing‐aged Black women are more likely than childbearing‐aged white women to have a low income and reside in impoverished neighborhoods that challenge their access to secure and safe housing, good jobs, good education, nutritious food, and safety. Respondents also indicated that Black women endured more daily stress resulting from structural discrimination in a variety of areas and interpersonal interactions.

Most respondents noted that problematic health‐related behaviors (nutritionally poor food choices, missed appointments) were rooted in social conditions and the debilitating effects of racism. However, 3 respondents attributed poor outcomes to genetic makeup or hereditary traits, and one respondent emphasized the nonadherence of individuals without noting contextual factors:

In my personal experience, the population I serve will not follow provider instructions on how to care for themselves while pregnant. I have had clients refuse to go to the hospital for treatment, they do not come to their appointments, and they will not change their diets. I serve a predominantly African American population on public assistance. There are resources in the area that they will not utilize. They want to be unhealthy and pregnant but then wonder why they get so sick.

Fifteen respondents argued that to decrease Black maternal mortality, society had to end white supremacy or structural racism. Others named specific social policy changes: safe and affordable housing, better education, better access to nutritious food, better working conditions, higher pay, and family and medical leave.

### Access to Health Care

Respondents attributed high Black maternal mortality and morbidity to lack of access to health care, including primary care. Several respondents suggested a system of universal health insurance, with one explicitly arguing that this should take the form of single‐payer public health insurance, with expansion of Medicaid and Special Supplemental Nutrition Program for Women, Infants, and Children until policymakers implement a single‐payer system. Many recommended Medicaid expansion to cover pre‐ and postnatal periods, care sites outside of hospitals, and more types of practitioners. Respondents emphasized preconception access and prenatal care. Others addressed better reimbursement for prenatal care and for more racially and occupationally diverse birth practitioners. Respondents noted that one cause of heightened Black maternal mortality and morbidity is conditions untreated before pregnancy.

Ensure they get health care earlier to identify any health issues that could impact a future pregnancy. Identify diabetes, high blood pressure, obesity, and infections prior to pregnancy, and focus on improving the health of women prior to conception. Educate women before pregnancy about risks and ways to reduce their risk.

Responses also captured the difficulty of timely and satisfactory entry into, and continued attendance at, prenatal care during pregnancy.

### Provider Practices

Respondents focused on deficiencies in the provision of health care for Black women, citing poor quality of care, disrespect of Black women, and poor communication between patient and provider. One respondent summarized the problems within the health care system:

Inequitable care given to women on Medicaid, poorer quality hospitals in poorer neighborhoods and in communities of color, judgement of women of color who have kids but aren't married, judgement of women of color who have kids but are young, poor treatment of childbearing women of color overall, the perception that women of color don't care about their health or the health of their babies, the assumption that women of color do drugs, drink and engage in risky behavior, the assumption that women of color don't get prenatal care, the assumption that women of color are all in ill health anyway, so they are going to have sicker babies. People don't listen or act when women of color express their concerns!!

Five subthemes related to improvements in provider practices emerged from the open‐ended questions: the perinatal workforce, modes of perinatal service delivery, interpersonal listening, evidence‐based perinatal practices, and midwives’ and other perinatal practitioners’ roles in the clinical setting.

### The Perinatal Workforce

The greatest number of responses to the open‐ended question about how to decrease Black maternal mortality named more training of providers to increase cultural sensitivity and reduce implicit racial bias. Many respondents urged more racial and ethnic diversity in the provider workforce. They commented about the cultural sensitivity and identity of the workforce and also identified the need for more midwives, particularly Black midwives, and doulas as a proportion of the perinatal care workforce.

We need active enrollment and encouragement of practitioners of color and community initiatives to increase brown and Black success. White practitioners should continue to discuss and explore implicit bias, discard the anti‐racism means being color blind paradigm, and recognize the unique health concerns and cultural approaches to health care in different communities.

### Modes of Perinatal Service Delivery

Respondents recommended reforming traditional models of perinatal health care and expanding innovative modes of care. They recommended slowing down conventional perinatal practice in traditional settings, with longer appointments allowing for whole person care. They recommended team‐based, multispecialty provider groups that would include not only midwives but also social workers, mental health workers, and nutritionists. Respondents recommended clinic‐based navigators to help patients make and keep appointments and ensure access to and regular use of prescribed medications.

Respondents also recommended innovative care models involving more midwife and doula care or new geographic locations and care institutions. Several recommended care teams or doula programs specifically directed toward Black women, like Birthing Beautiful in Cleveland, a grassroots organization that provides services for pregnant Black women living in underserved communities.^20^ Several respondents recommended improving geographic accessibility, through such measures as home visits and group prenatal care in centrally located public buildings like libraries, churches, and municipal buildings. Respondents also suggested more freestanding community‐based birth centers in or close to Federally Qualified Health Centers and friendly to low‐income women of color.

Respondents noted the importance of Medicaid coverage and adequate reimbursement for innovative forms of care. They pointed out, for example, that despite data from Strong Start showing that birth center care is highly effective at lowering the morbidity rate for African American women,^21^ birth center care may not be available in some states and localities.

Increase insurance reimbursement for prenatal care so that providers can spend more time with patients and so more RNs can be hired for care coordination. Obstetrics has historically been a very challenging financial picture, and providers are constantly being pressured to do more with less time and funds. This is in contrast to other disciplines; for example, an anesthesiologist may bill more for one epidural analgesia placement than we receive for our global package of all prenatal care plus birth plus postpartum care combined.

### Interpersonal Listening and Attention to Individual Clients

Responses highlighted the importance of increased respect for and listening to women from all perinatal health practitioners. Respondents thought that deeper listening and more individual attention would result if there were higher nurse‐to‐patient ratios, an emphasis on quality of care not number of encounters, and longer appointments that allowed for whole person care.

### Evidence‐Based Perinatal Practices

Many respondents called for better national and local data about maternity outcomes and racial disparities. They recommended national standards of care based on evidence. Few references were made to the already existing patient safety bundles issued by the National Partnership for Maternal Safety under the guidance of the Council on Patient Safety in Women's Health Care.

Standardized care practices in maternity care would leave less room for implicit bias in care decisions. There must then also be feedback and quality improvement initiatives to assess how a given institution is doing. Hold health institutions accountable to evidence‐based care practices and protocols.

### Midwives’ and Other Perinatal Practitioners’ Roles in the Clinical Setting

Respondents identified ways in which they, through their own clinical practices, could address Black maternal mortality. They noted their own roles within their institutions in creating wraparound services for transportation, peer support, and mental health services and in improving clinic scheduling flexibility to facilitate access. One cited the importance of establishing basic social needs screening and subsequent referral and monitoring: “Early intervention to look at social factors for individual client, including housing, family support, food access, providing referrals and follow‐ups.” Respondents noted they could work to mandate safety bundles, establish and serve on maternal review boards, create community teams of physicians, CNMs/certified midwives (CMs), and doulas. Respondents noted that they themselves could undergo implicit bias training and encourage awareness among provider colleagues. They encouraged midwifery clinical practices, including listening to and believing clients as they reported symptoms, educating clients about warning signs, supporting stress reduction, and generally explaining healthy pregnancy. Respondents suggested that midwives need to be advocates and activists within health care institutions and communities to increase awareness, promote change, and develop programs to support women in general and particularly women of color. A few respondents talked about building support groups and networks for women to seek the resources they needed.

Midwives and others who address maternal mortality with patients noted that this conversation did and could address both social and health factors influencing pregnancy outcomes, create a communication partnership, acknowledge burdens imposed by racism, and empower the patient to ask questions and express concerns. Practitioners who did not address maternal mortality with patients said that conversations with individual patients could not alter the powerful institutional and social systems affecting patient health and health care. One participant wrote: “Individual practitioners don't make the practices rules. If practices don't make an effort to accept and help patients with financial challenges, then the mortality and morbidity will continue to climb.” Additional quotes are available online in Supporting Information: Appendix S1.

## DISCUSSION

This exploratory study offers important information about midwives’ and other perinatal health providers’ analysis of, experience of, and recommendations regarding Black maternal mortality, areas not yet explored in the research literature. Although existing work on midwives’ perceptions has looked at discrete perinatal practices, this project identified midwives’ perspectives on a critical social and health issue—excessive Black maternal mortality—using concepts of social determination of health, increasingly used to examine racial and class health disparities and health outcomes generally. Both in naming causes and proposing solutions, perinatal health workers addressed all 3 levels of determination: primary social determinants, health care access, and provider practices.

A large majority of respondents, most of whom were white midwives, were aware of excessive Black maternal mortality. Although a majority said they had witnessed discrimination based on race and class in their professional setting, the majority held that it was important for practitioners to respect people's diverse social identities, attend to all clients’ physical and emotional health, and allow all women to have meaningful input into decisions about their care and childbirth.

Responses paralleled the existing literature in emphasizing primary social determinants of Black maternal morbidity and mortality, not only in erecting social barriers^7^, ^8^ but also in creating physiologic impacts through elevated allostatic load due to institutional exclusion and interpersonal discrimination.^9^, ^22^ Responses mirrored the literature in noting that Black maternal morbidity and mortality are related not only to primary social determinants but also to lack of access to primary and prenatal care. The health status of minority women with low income contributes to persistent, and sometimes increasing, disparities in birth outcomes.^23^, ^24^ Research has shown that expansion of general Medicaid, Medicaid pregnancy coverage, or coverage generally could improve some chronic conditions and maternal health.^25^, ^26^, ^27^, ^28^ However, respondents, unlike much recent literature, did not emphasize the importance of postpartum processes, ideally integrated into prenatal counseling and facilitated by extended postpartum insurance coverage and paid family leave.^29^, ^30^ Postpartum coverage is particularly important for perinatal mental health conditions.^31^, ^32^ The research literature, similar to the respondents, also identifies other access barriers: challenges of getting to clinics, receiving reasonably prompt attention in clinics, and adhering to prenatal visits and best practices while coping with everyday life stresses and lack of access to health care providers of choice.^33^, ^34^, ^35^, ^36^

Racialized practices inside health care institutions and corresponding improvement measures are also well documented in the research literature. The majority of CNMs/CMs in the United States identify as white (84.9%). Midwives identifying as Black or African American make up 7.3%, and those who identifying as Latino make up 5%.**^37^** Research has documented the low proportion of midwives among perinatal health workers in the United States, a lower proportion than in other high income democracies, despite positive outcomes of midwifery care.^38^ The problem of racial incongruence between patients and providers is twofold: a predominantly white workforce engenders mistrust among many Black women, impeding health care use, and white practitioners may not fully respect or understand women of color.^39^, ^40^ Survey responses indicated complex thinking about the personnel mix that might reduce Black maternal mortality. Although respondents note midwifery's approach to the whole person is essential and that racial and ethnic identity may be critical to deep understanding of and trust by patients, they maintain that all providers have the potential to provide high quality care to Black women. Additionally, survey responses were consistent with research noting that practitioners often fail to listen to women; insufficiently rigorous health protocols allow discretionary inequitable treatment; practitioners fail to attend to more complex social and medical cases; and Black women receive care at sites delivering lower quality care.^41^, ^42^, ^43^, ^44^

### Implications for Practice, Policy, and Research

In referring to their own roles as perinatal health workers, respondents cite a range of care roles addressing both social determinants and biological determinants of maternal health. These correspond to those roles identified in *The Future of Nursing 2020‐2030*, which focuses upon centering health and health care equity.^45^ Thus, many midwives and other perinatal health workers, focused on holistic patient health and therefore their social context as well as their medical needs, seem to already be practicing for health equity in the ways recommended by the National Academy.

Based upon their holistic approaches to patients and understanding of their clients’ everyday lives, midwives may be positioned to exercise leadership in health care teams to improve provider practices, including adoption of more client‐centered practices and safety bundles. The clinical practice of screening for social determinants and connecting clients to community resources should be consistently implemented. Midwives and other perinatal health workers may want to seek additional ways to integrate mental health screening and interventions, as well as postpartum planning, into prenatal care.

Few respondents explicitly embrace public advocacy roles in working to improve perinatal health, but this may be in part an effect of the framing of the research in terms of practitioner roles or because practitioner responsibilities are already overwhelming. However, midwives may also want to think of themselves as midwife‐citizens and engage in politics and policy advocacy that extend beyond clinical practice issues to the intermediary social determinant of health care access. Perinatal health workers can support the expansion of Medicaid to all low‐income adults or the expansion of Medicaid pregnancy coverage from 60 days to one year postpartum. In addition, they can advocate for public policies that improve primary social determinants of health: systemic racism, social inequality, poverty, housing insecurity, and more.

This research employed a one‐time online survey with numerous limitations. This exploratory survey was launched without tests to define reliability or validity. It did not employ a stratified sampling technique and used convenience sampling. It was an initial attempt to explore a broad set of questions about perinatal health workers’ awareness, analysis, observations, and recommendations regarding excessive Black maternal mortality. The survey explicitly named excess Black maternal mortality as its topic and invited voluntary participants, who may have been more informed and engaged in this problem than other midwives and perinatal health workers who did not participate. Unfolding public events (a series of police killings of Black men, a disputed election, and media representations of these events) may have affected survey responses, recorded in late 2019. In addition, the survey did not associate responses with the racial and ethnic identity or other specific characteristics of respondents. Because perceptions of racism vary by race,^46^ maternity care policies such as insurance and family leave vary by state, pregnancy and birth resources vary by location,^47^ and institutional settings vary, such identifiers in future research might be helpful. In addition, it would be interesting to compare the responses of midwives to those of other perinatal health providers, especially obstetricians. Further quantitative and qualitative research might also explore the discrete themes that emerged in the analysis.

Overall, the research findings confirm that midwives and other perinatal health practitioners have a comprehensive view of causes and potential remedies for excessive Black maternal mortality. The responses show that midwives and others, positioned between health care institutions and their clients’ everyday lives in communities, see the Black maternal mortality crisis as generated both by broad social and political factors, including structural racism, and by problematic and often racialized practices in health care institutions. Similarly, to reduce Black maternal mortality, perinatal health workers recommend actions ranging from policies addressing broad social determinants to specific steps perinatal health workers could take as providers. Midwives and others see both causes and solutions as complex and multidimensional, matching recognition of plural causality in the research literature.

## CONFLICTS OF INTEREST

The authors have no conflicts of interest to disclose.

## Supporting information


**Appendix S1**. Emerging themes and supporting quotations: Attributes across levels of determinants.
