# Supplementary material for: Midwives’ and Other Perinatal Health Workers’ Perceptions of the Black Maternal Mortality Crisis in the United States
Source: J Midwifery Womens Health. 2022 Nov 22;68(1):62–70. doi: 10.1111/jmwh.13433 (PMC10099225; doi:10.1111/jmwh.13433)
Supplement: Supplementary file 1 — Appendix S1. Emerging themes and supporting quotations: Attributes across levels of determinants. [file JMWH-68-62-s001.docx]

**Supporting Information: Appendix S1**

**Emerging themes and supporting quotations: Attribution across levels of determinants**

| **Primary social determinants** | “Racism in American society and within healthcare institutions/providers, chronic stress, transgenerational stress, lack of access to high quality care increase the negative impact of other social determinants of health including poverty, lack of access to health foods, health care illiteracy, etc.”  “Bias, racism, and poverty impact individuals. The percentage of people of color living in poverty is higher and disproportionately more than the percentage of white, non-Hispanic individuals. Poverty contributes to a multitude of health complications not faced by those who can afford healthy food, gym memberships, and regular medical care.”  “Racism. At the root of it all. Anyone who says it is genetic or because Black women have poorer health needs to be reminded of structural and systemic factors that cause not only poor health but even genetic changes. It makes me think of Geronimus’ ‘weathering hypothesis’ and transgenerational, epigenetic health.”  “Raise the minimum wage to $15/hour, improve access to housing, increase delivery of healthy food in neighborhoods—vegetable and fruit trucks. “ |
| --- | --- |
| **Access to health care** | “We need universal health care so that women can receive care when they are and aren’t pregnant. Expand access to mental health care and social services so we can treat women as a whole person instead of treating one body system at a time.”  “Increase health insurance coverage for all pregnant women.”  “Easier process to access Medicaid during pregnancy.”  “Higher Medicaid reimbursement rates, improved access to high-quality care by diminishing structural challenges, holding health institutions accountable to evidence-based care practices and protocols.”  “Staff assistance with insurance issues, support and increased number of social workers to address home issues, vouchers for transportation/mobile units for home visits. . .”  “More clinics, more CNMs in areas heavily populated by African American women and families. This is one symptom of the overall disease of systemic racism; people need to believe that Black lives matter.” |
| **Provider practices** |  |
| **Workforce** | “Implicit bias training for all health care providers and health care administrators, as well as nursing and medical students. I would introduce anti-racism training into every level of worker in every hospital and teach it in midwifery programs, nursing programs, and medical residents.”  “As midwifery care has shown improved outcomes, increasing access to midwifery care is an evidence-based strategy to address the problem. Recruiting and supporting care providers who belong to minority populations will promote trust, credibility, and authentic perspective to our health care system. There is also evidence this translates into improved outcomes.”  “The increase in non-white primary care providers is important. Women need to see and trust care providers who look like them and understand their needs in a way even the most compassionate white provider can’t. The growth in midwifery will play a key role in addressing the needs of women one-to-one, no matter her race or ethnicity.” |
| **Modalities of service delivery** | “Greater ease of access to a team-based, multi-specialty provider group including social workers, mental health caregivers and nutritionists.”  “A clinical navigator to make sure patients have and keep appointments and provide means for them to get there, funds to get supplies and medications. A nurse navigator to follow up on clinical issues. An educational coordinator for prenatal classes (centering), diabetes classes, HTN classes. Funding this.”  “Remove all barriers to birth center care (Michigan neither  licenses birth centers nor does its Medicaid pay for birth center care, nor can a CNM own a birth center in Michigan). The data from Strong Start show that birth center care is highly effective at lowering the morbidity rate for African American women.”  “Perinatal doula programs created by and for Black women, like Birthing Beautiful in Cleveland.”  “Fostering RN visit programs during pregnancy and postpartum.”  “Group pre-natals, prenatal care provided in remote locations utilizing public buildings like libraries, churches, municipal buildings more centrally located than the doctor’s office; go to the population having the most difficulty getting adequate care.” |
| **Evidence-based care** | “Standardized care practices in maternity care would leave less room for implicit bias in care decisions. Then there must also be feedback and quality improvement initiatives to assess how a given institution is doing.”  “For too long obstetrics has practiced in the grey area where women are victim to old habits and biased attitudes. The lack of experimental research due to the nature of risk for research on pregnancy and infants has created a laissez faire attitude towards evidence. It often seems like the new evidence-based information does not trump the way certain providers practice. Most of the time, this attitude is coming from the top and trickling down to new providers. Additionally, when poor outcomes exist due to no-evidence-based practices and biased attitudes, they are rarely addressed.”  “Following evidence-based guidelines is a place to start—the bare minimum. . . From that base, we then individuate the care as it relates to who the woman is and what her risks might be, etc.” |
| **Listening to clients** | “People don’t listen or act when women of color express their concerns. They assume that women of color are all in ill health anyway, so they are going to have sicker babies. They judge women of color who have kids but aren’t married or are young. The perception that women of color don’t care about their health or the health of their babies. The assumption that women of color do drugs, drink and engage in risky behavior.”  “Do a better job educating providers about listening to patients versus ordering a plethora of tests and going on a fishing expedition. Also encourage all women to take responsibility for their health care decisions and advocate for themselves.” |
| **Midwife/perinatal practitioner clinical practices** | “Educate about what is normal, so that women are not afraid of normal birth. Educate on the dangers of pregnancy, warning signs, when to see a provider ASAP. Listen and believe when they report symptoms. Be aware of resources and readily refer and connect patients.”  “I focus on their successes, all the difficulties they have surmounted, all the strengths they have used to overcome their difficulties. I offer education on self-care, self-valuing, healthy and life-affirming practices.”  “Survey own implicit bias, anti-racism and bias training, training on effects of racism, CEU’s on how to listen better, acceptance of white privilege, watch WOC closely.”  “Intentionally ask about domestic violence.”  “Mindfulness techniques as part of prenatal care.”  “Support a clinical navigator to make sure patients have and keep appointments and provide means for them to get there, funds to get supplies and medications. A nurse navigator to follow up on clinical issues. An educational coordinator for prenatal classes (centering), diabetes classes, HTN classes. Using other navigators.”  “Close follow-up postpartum and home visits by nurse or provider on prescribed days postpartum.” |
